# The Antitriple Negative Breast cancer Efficacy of *Spatholobus suberectus* Dunn on ROS-Induced Noncanonical Inflammasome Pyroptotic Pathway

**DOI:** 10.1155/2021/5187569

**Published:** 2021-10-06

**Authors:** Feng Zhang, Qingqing Liu, Kumar Ganesan, Zeng Kewu, Jiangang Shen, Fang Gang, Xiaohe Luo, Jianping Chen

**Affiliations:** ^1^School of Chinese Medicine, Li Ka Shing Faculty of Medicine, The University of Hong Kong, 10 Sassoon Road, Pokfulam, Hong Kong, China; ^2^Shenzhen Institute of Research and Innovation, The University of Hong Kong, Shenzhen, China; ^3^School of Pharmaceutical Sciences Peking University Health Science Center, Peking University, Peking, China; ^4^Guangxi Key Laboratory of Applied Fundamental Research of Zhuang Medicine, Guangxi University of Chinese Medicine, Nanning, China; ^5^The Center of Clinical Research of Endocrinology and Metabolic Diseases in Chongqing and Department of Endocrinology, Chongqing University Three Gorges Hospital, School of Medicine, Chongqing University, Chongqing, China; ^6^Department of Laboratory Medicine, Chongqing University Three Gorges Hospital, School of Medicine, Chongqing University, Chongqing, China

## Abstract

Breast cancer (BCa) is the leading cause of women's death worldwide; among them, triple-negative breast cancer (TNBC) is one of the most troublesome subtypes with easy recurrence and great aggressive properties. *Spatholobus suberectus* Dunn has been used in the clinic of Chinese society for hundreds of years. Shreds of evidence showed that *Spatholobus suberectus* Dunn has a favorable outcome in the management of cancer. However, the anti-TNBC efficacy of *Spatholobus suberectus* Dunn percolation extract (SSP) and its underlying mechanisms have not been fully elucidated. Hence, the present study is aimed at evaluating the anti-TNBC potential of SSP both in vitro and in vivo, through the cell viability, morphological analysis of MDA-MB-231, LDH release assay, ROS assay, and the tests of GSH aborted pyroptotic noninflammasome signaling pathway. Survival analysis using the KM Plotter and TNM plot database exhibited the inhibition of transcription levels of caspase-4 and 9 related to low relapse-free survival in patients with BCa. Based on the findings, SSP possesses anti-TNBC efficacy that relies on ROS-induced noncanonical inflammasome pyroptosis in cancer cells. In this study, our preclinical evidence is complementary to the preceding clinic of Chinese society; studies on the active principles of SPP remain underway in our laboratory.

## 1. Introduction

There are seldom chemotherapeutic medications that can gain moderate success in triple-negative breast cancer (TNBC) management. Breast cancer (BCa) is the leading cause of women's death worldwide. Among them, TNBC accounts for almost 10-15% of all BCs, which refers to the absence of estrogen and progesterone receptors and overexpression of human epidermal growth receptor 2 [1, 2]. It is one of the most troublesome subtypes of BCa because of its easy recurrence and highly aggressive properties. Although some basic investigations related to immunotherapy and targeted therapy have shown great potential to inhibit the development of cancer [3, 4], they still seldom chemotherapeutic drugs, which can offer positive pathologic complete response for the management of TNBC in the clinic [5].

*Spatholobus suberectus* Dunn (SSD, Leguminosae), documented as a traditional Chinese medicine (TCM) named “Ji Xue Teng,” has been used in the clinic of Chinese society for hundreds of years in hematopoiesis and applied to treat rheumatism, anemia, and menoxenia [6]. SSD has widely been used in conventional medicines that possess various pharmacological activities, viz., antioxidant, antimutagenic, antiplatelet, immunomodulatory, antibacterial, antiviral, neuroprotection, and blood circulation improvement [7–12]. SSD comprises several bioactive compounds in which flavonoids are predominant, including 3′,4′,7-trihydroxyflavone, 3′-hydroxy-8-methoxyvestitol, butin, calycosin, dihydrokaempferol, dihydroquercetin, eriodictyol, liquiritigenin, plathymenin, and prunetin, and many of them exert anticancer properties [11, 13, 14]. Furthermore, numerous experimental pieces of evidence have been shown that crude extracts of SSD produce a favorable outcome in the management of cancer [15–17] and coronary heart diseases [18]. SSD has been used in the clinic of TCM as a potential drug to treat BCa patients and accounts for excellent response [19]. SSD has potent anticancer effects on BCa with the capacity of causing apoptosis and obstructing cell cycle, LDH, and BCa migration via the MAPK PI3K/AKT pathway [20, 21]. However, the anti-TNBC efficacy of SSP and its underlying mechanism has not been fully elucidated.

Earlier researchers paid great attention to investigating programmed cell death: apoptosis, which is believed not to trigger inflammation [22]. It is considered an essential component of several processes such as normal cell turnover, growth, the function of the immune system, embryonic development, and chemical-induced cell death [23]. Recently, pyroptosis is another kind of programmed cell death, which is varied in the mechanisms of apoptosis, and proved to be crucial for clearing dangerous infections [24, 25]. Pyroptosis is generally lytic cell demise accompanied by rapid cell membrane rupture [26], in which pores are initially formed in the membrane of the cell, causing water influx and cell swelling, causing cell-membrane damage. Hence, pyroptosis is believed to be more inflammatory and immunogenic than apoptosis [27]. Several pilot studies have also clarified that pyroptosis may trigger inflammation and recruit immune cells to the pyroptotic area [28, 29]. Currently, mounting research related to pyroptosis is extensively studied in cancer.

The relationship between ROS and tumor cell pyroptosis has been well established [30]. Low doses of ROS normally stimulate cell proliferation in a wide variety of cancer cell types [31, 32]. However, elevated ROS triggers tumor cell pyroptosis-dependent caspases [33]. Some chemotherapeutic drugs are addressed to induce tumor cell pyroptosis dependent on caspase-3 [34, 35]. Mechanistically, there are two different kinds of signaling pathways involved in pyroptosis. The first one is the caspase-1-dependent process, named “canonical” inflammasome activation, which is mediated by a dynamic mediator, gasdermin D (GSDMD) [36]. The dynamic caspases-1, -4, -5, and -11 generally cleave GSDMD within a linker between the domains of amino and carboxy-terminal. After the breakdown, the N-terminal generates pores in the cell wall to cause pyroptosis resulting in transmembrane ion flux, cytoplasmic swelling, and osmotic lysis [37]. Secondly, a “noncanonical” inflammasome activation has been termed as a pathway that is ROS/caspases axis-dependent, which is also mediated by GSDMD or/and gasdermin E (GSDME) [38–41]. Nevertheless, there are seldom reports on TCM that can trigger pyroptosis in cancer management. Therefore, the present study is aimed at evaluating the anti-TNBC efficacy of SSP on TNBC cell lines by analyzing cellular characteristics including cell viability, cell morphology changes, LDH release assay, ROS assay, and glutathione (GSH) aborted pyroptotic noninflammasome signaling pathway.

## 2. Materials and Methods

### 2.1. Preparation of Spatholobus suberectus Percolation (SSP) Extract

SSP extract was prepared in accordance with EMA guidelines as described previously with some modifications [42]. Briefly, dried SSD stems, which were provided by Guangdong Kangmei Pharmaceutical Co., Ltd. (Guangdong Province, China), were ground into coarse powder, and it was extracted using a percolating device with 10 times volume (v/w) of 60% ethanol. The filtrate was then concentrated under reduced pressure by a rotary evaporator (IKA RV 10, IKA- Werke GmbH & Co. KG, Darmstadt, Germany). The obtained percolation powder was then freeze-dried (percent yield 20%) and stored at 4°C for further use.

### 2.2. UHPLC Analysis

The ultra high-pressure liquid chromatography (UHPLC) analysis was conducted with an Ultimate 3000 system (Thermo Scientific, MA, U.S.A.) equipped with a quaternary pump, a vacuum degasser, an auto-sampler, and a DAD UV-Vis detector. The gradient elution was composed of solvent A (water: 0.3% formic acid, v/v) and solvent B (acetonitrile). A reverse-phase packing C_18_ column (100 mm × 2.1 mm, 4 *μ*m, ACE, UK) was used in this experiment. The mobile phase condition was performed as follows: 0 min, 2% (B); 2 min, 2% (B); 5 min, 10% (B); 12 min, 10% (B); 20 min, 20% (B); 25 min, 20% (B); 26 min, 25% (B); 32 min, 35% (B); and 37 min, 40% (B). The flow rate was 0.4 ml/min, and the injection volume was 5 *μ*L. The experiment was operated at 30°C, and the detection wavelength was 280 nm. All solutions were prepared with 0.22 *μ*m filtration (Sigma-Aldrich, St. Louis, MO, USA) for the samples. The mobile phase was purged before injection of UHPLC. Catechin (Sigma-Aldrich), procyanidin B2 (Sigma-Aldrich), epicatechin (Sigma-Aldrich), genistein (Sigma-Aldrich), formononetin (Sigma-Aldrich), and SSP were accurately weighed and then dissolved in methanol. Identification was achieved by comparing retention times (RT).

### 2.3. Cell Culture and Treatment

MDA-MB-231, 4 T1, and BT 549 cells were obtained from American Type Culture Collection (ATCC, Manassas, VA, USA). All cells were maintained in Roswell Park Memorial Institute (RPMI) 1640 (Gibco, Grand Island, NY, USA.) or glucose-containing (4.5 g/L) Dulbecco's Modified Eagle Medium (DMEM, Gibco, Grand Island, NY, USA) according to the protocol of ATCC, which were supplemented with fetal bovine serum (FBS, 10% v/v, Gibco, Grand Island, NY, USA), penicillin (Sigma-Aldrich, St. Louis, MO, USA, 100 U/ml), and streptomycin (Sigma-Aldrich, St. Louis, MO, USA, 100 *μ*g/ml) in a humidified atmosphere of 5% CO_2_ at 37°C. Cells were seeded onto 96-well plates at the density of 3 − 5 × 10^3^/well. After undergoing serum starvation for 24 h, they were treated with different concentrations of SSP (200, 100, 50, 25, 12.5, 6.25, 3.13, 1.56 *μ*g/ml) or docetaxel (Beijing Aosaikang Pharmaceutical Co., Ltd, Jiangsu, China; 500, 250, 125, 62.5, 31.25, 15.63, 7.81, 3.91 *μ*g/ml). The tumor cell growth inhibitory effect of SSP or docetaxel on MDA-MB-231 and BT 549 was tested the cell viability and proliferation using CellTiter 96® AQueous Non-Radioactive Cell Proliferation Assay containing 3-(4,5-dimethylthiazol-2-yl)-5-(3-carboxymethoxyphenyl)-2-(4-sulfophenyl)-2H-tetrazolium) (MTS) kit (Promega, Wisconsin, U.S.A.) as per the manufacturer's protocol. The IC_50_ values of drugs for different cell lines were calculated by linear or nonlinear regression.

### 2.4. Determination of Intracellular ROS

For the determination of intracellular ROS, a chloromethyl derivative of 2′,7′-dichlorofluorescein (CM-H2DCFDA, Cat# C6827, Sigma-Aldrich, St. Louis, MO, USA) and Dihydroethidium (Cat# D11347, Sigma-Aldrich, St. Louis, MO, USA) staining were used to determine hydroxyl, peroxyl, and other cellular ROS activities. 2′,7′-dichlorofluorescein and dihydroethidium were used to determine the generation of cell cytosolic hydrogen peroxide and superoxide, respectively, which were analyzed according to the manufacturer's protocol. Briefly, MDA-MB-231 cells (2 × 10^5^ cells/ml) were treated with various concentrations of SSP (25, 50, 100 *μ*g/ml) for 24 h, followed by staining with CM-H2DCFDA, and dihydroethidium (20 *μ*M) was kept at room temperature for 45 minutes. Then, the cells were visualized under fluorescence microscopy.

### 2.5. Lactate Dehydrogenase Release Assay

Lactate dehydrogenase (LDH) is a cytoplasmic enzyme that discharges into the culture medium during the cell getting rupture. The release of cytoplasmic enzymes is generally taking place due to inflammation (pyroptosis), recognized as an indicator of cell membrane damage. The leakage of the enzyme was determined in the culture medium using a CytoTox 96 Non-Radioactive Cytotoxicity Assay Kit (Promega, Wisconsin, U.S.A) as per the manufacturer's instructions. Absorbance at 490 nm was measured using a BioTek Synergy 2 microplate reader (BioTek, Winooski, VT, USA). The LDH release levels were calculated according to the formula:

(1) $$\begin{array}{c} \text{LDH release level}=100\%\times \left.\left(\;\frac{\text{Test OD}-\text{Blank OD}}{\text{Vehicle control OD}-\text{Blank OD}}\right.\right) \end{array}$$

### 2.6. Morphological Analysis by Scanning Electron Microscopy

Morphological analysis was performed as described earlier [30]. Cells were treated with SSP (100 *μ*g/ml) for 24 h and were fixed with 2.5% glutaraldehyde (Sigma-Aldrich, St. Louis, MO, USA) overnight. The cells were rinsed thrice using phosphate buffer saline, and the critical point of the drying procedure was carried out. Samples were dehydrated through a graded series of ethanol (30, 50, 70, 95, and 100%) and dried in a Critical Point Dryer using liquid carbon dioxide. The dried specimens were mounted on specimen holders (aluminium stubs) for scanning electron microscopy (SEM), using double-sided adhesive tape, glue, colloidal silver, or colloidal carbon. Then, a thin layer (100-200 Å) of the metallic film was coated on the specimen surface for electrical conduction using either a sputter coater or a vacuum evaporator. Gold, gold-palladium, platinum, aluminium, or carbon was commonly used for the preparation of the thin conducting film. Image with a Hitachi S-3400 N scanning electron microscope was operated for the present study at 20 kV.

### 2.7. Animals

Female (BALB/c) nude mice (6-7 weeks old) were purchased from Harlan Laboratories, Indianapolis, IN, USA, that were housed and maintained in Laboratory Animal Unit, the University of Hong Kong, a specific pathogen-free and climate-controlled room (22 ± 2°C, 50 ± 10% relative humidity) with a 12 h light/dark cycle and provided with diet and water ad libitum. The xenograft assay was performed as described before with some modifications [43, 44]. MDA-MB-231 cells (2 × 10^6^/site) were implanted subcutaneously into the bilateral flank of each mouse. Palpable and measurable tumors were initially found 10 days after cell injection. Then, the animals were randomly assigned into four groups that were receiving the following treatments: the vehicle control group (*n* = 5) received Milli-Q water; the SSP-L group (*n* = 5) received SSP (0.4 g/kg/p.o, daily); the SSP-H (*n* = 5) group received SSP (0.8 g/kg/p.o, daily); the DTX group (*n* = 5) received docetaxel (5 mg/kg/i.p. week). The tumor size was calculated using the formula: 0.5 × length×width2. All experiments were approved by the Institutional guidelines of Laboratory Animal Care and *Committee on the Use of Live Animals in Teaching and Research* (CULATR No.: 4484-17).

### 2.8. Acute Toxicity Study

Acute toxicity studies were performed to determine the short-term adverse effects of a drug when administered in a single dose or multiple doses during 24 hours in two rodent species. The acute oral toxicity study was evaluated as per OECD guidelines. The studies were carried out in *BALB*/*c* mice (20–30 g) and Sprague-Dawley rats (150-180 g), respectively, using a single dose or multiple doses, which were treated orally. Thirty animals, divided into respective 5 groups, were designed for the study of acute toxicity via the oral route. Each group contains 6 animals (3 males and 3 females) receiving a single oral dose of 2, 4, 8, and 10 g/kg body weight of SSP extract, while the control group was administrated with distilled water. The general behavior of the animal and signs of toxicity were observed continuously for 1 h after the oral administration and then intermittently for 4 h and thereafter for 24 h. The animals were further observed once a day up to 14 days following treatment for behavioral changes and signs of toxicity and/or death and the latency of death. The LD_50_ value was determined according to the method described by Kharchoufa et al. [45].

### 2.9. Western Blot Analysis

Western blot assay was conducted as previously described [17]. The proteins from the cell were lysed in RIPA buffer (pH = 7.4) comprised of protease inhibitors cocktail (10 *μ*g/ml, Cat# 5872S, Cell Signaling Technology, MA, U.S.A.). The contents were centrifuged at 12,000 g at 4°C for 20 min, and the concentration of protein in the supernatants was determined using Bradford reagent (BioRad) with bovine serum albumin (BSA, Sigma Aldrich, St. Louis, MO, U.S.A.) as the standard. The protein samples were separated by electrophoresis on SDS-PAGE 10% or 12.5% gels. After blocked in 3% BSA, the membrane was incubated with primary antibodies, GAPDH (Cat# 2118 s, Cell Signaling Technology), caspase-1 (Cat# sc-56036, Santa Cruz Biotechnology, U.S.A.), GSDMD (Cat# 93709 s, Cell Signaling Technology), JNK1/2/3 (Cat# YT2440, Immunoway, TX, U.S.A.), caspase-3 (Cat# sc-7148, Santa Cruz Biotechnology), caspase-9 (Cat# 9502, CST), caspase-4 (Cat# ab238124, Abcam, Cambridge, United Kingdom), cleaved caspase-3 (Cat# 9661 s, Cell Signaling Technology), and GSDME (Cat# ab215191, Abcam) as needed. For secondary antibodies, antibodies to mouse (Cat# 7076, Cell Signaling Technology) and rabbit (Cat# 7074, Cell Signaling Technology) were used. To visualize protein bands, a chemiluminescence (ECL) system (Cat# WBLUF0500, Millipore, MA, U.S.A.) was used.

### 2.10. Collection and Analysis of Biological Information

The association between caspase-4, caspase-9, and overall survival was performed by the online tool KM plot (http://kmplot.com/) [46] with the Affymetrix ID: 213596_at and 237451_x_at, respectively. Differential gene expression analyses of the tumor, normal, and metastatic tissues were conducted by the online tool TNMplot (https://www.tnmplot.com/) with the genes' symbols based on RNA-Seq data offered by the database [47].

### 2.11. Statistical Analysis

Nonlinear regression was operated with GraphPad Prism 7 (GraphPad Software, San Diego, CA, USA) choosing log(inhibitor) vs. response—variable slope (four parameters) as the equation. All data were expressed as mean ± standard deviation. Tukey's multiple comparison test was carried out on data from at least three independent experiments. The differences between the two groups were performed using two-tailed Student's *t*-test, and significance was established at *p* ≤ 0.05.

## 3. Results

### 3.1. UHPLC Analysis of SSP

**Figure 1:**
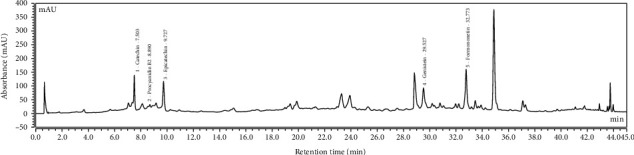
Identification of active constituents from SSP by UHPLC analysis.

**Figure 2:**
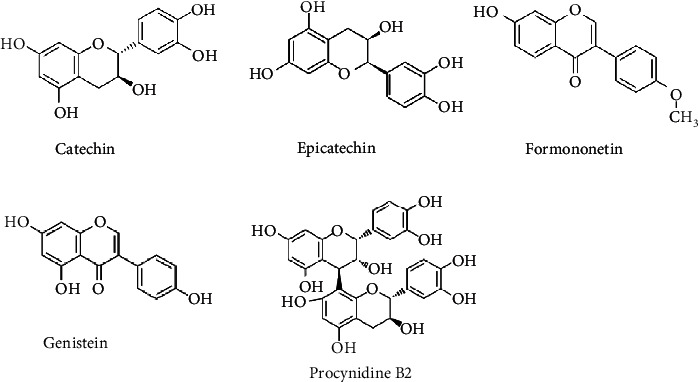
Structures of isolated compounds from SSP.

A simple and speedy UHPLC method was used to determine the major constituents (catechin, procyanidin B2, epicatechin, genistein, and formononetin) that appeared in SSP. The constituents were separated on reverse-phase-C_18_ column developing a mobile phase comprised of formic acid (0.05%) in acetonitrile in the gradient elution mode. Under these conditions, flavonoids and isoflavones were separated in a 45 min run. The standard peaks 1-5 were identified as catechin (RT, 7.503 min), procyanidin B2 (RT, 8.690 min), epicatechin (RT, 9.727 min), genistein (RT, 29.527 min), and formononetin (RT, 32.773 min) (Figure 1) according to the retention time (RT) and UV-Vis spectra of the standards. The outcomes demonstrated that SSP contained five compounds (Figure 2). The analyses were repeated thrice and verified the constituents.

### 3.2. Growth Inhibitory Efficacy of SSP on Cancer Cell Lines

**Figure 3:**
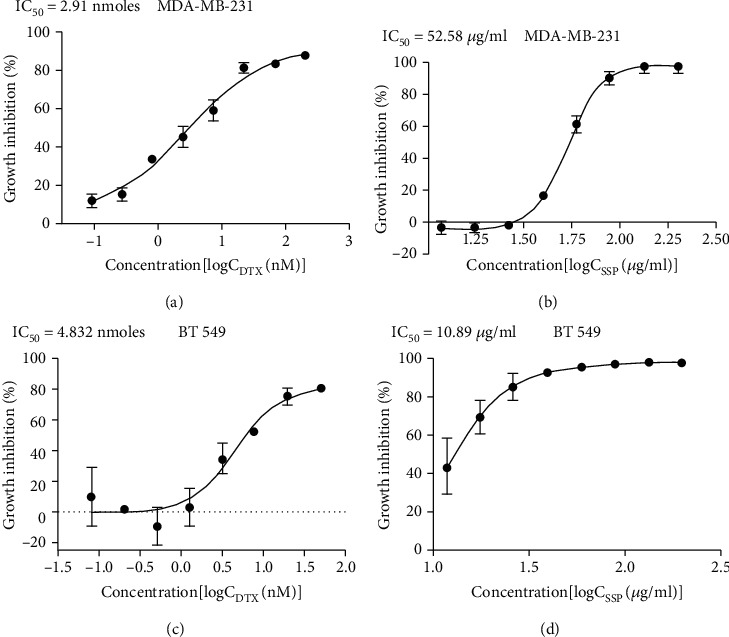
(a–d) Growth inhibitory efficacy of SSP and DTX on MDA-MB-231 and BT 549 cell lines.

The growth inhibitory efficacy (IC_50_) of SSP was tested in the cell lines of MDA-MB-231 and BT 549 cells with the positive control (DTX) in vitro. The cells were treated with different concentrations of SSP (200, 100, 50, 25, 12.5, 6.25, 3.13, 1.56 *μ*g/ml) or DTX (500, 250, 125, 62.5, 31.25, 15.63, 7.81, 3.91 nmoles), and growth inhibition curves were presented in Figures 3(a)–3(d). Through nonlinear regression, the IC_50_(s) of SSP and DTX were prepared. For MDA-MB-231 cells, IC_50_ of DTX and SSP was 2.91 nmoles and 52.58 *μ*g/ml, respectively. Similarly, for BT 549 cells, IC_50_ of DTX and SSP was 4.832 nmoles and 10.89 *μ*g/ml, respectively. SSP and DTX exerted significant growth inhibition effects (IC_50_) based on the increasing concentration exhibited in both cancer cell lines. The growth inhibition efficacy of SSP occurred significantly at lower concentrations in BT 549 and higher concentrations in MDA-MB-231. Moreover, DTX had growth inhibitory effects at minimum concentrations in MDA-MB-231 and maximum concentration in BT 549 (Figures 3(a)–3(d)).

### 3.3. Determination of Growth Inhibitory Efficacy of SSP in Xenograft Animals

**Figure 4:**
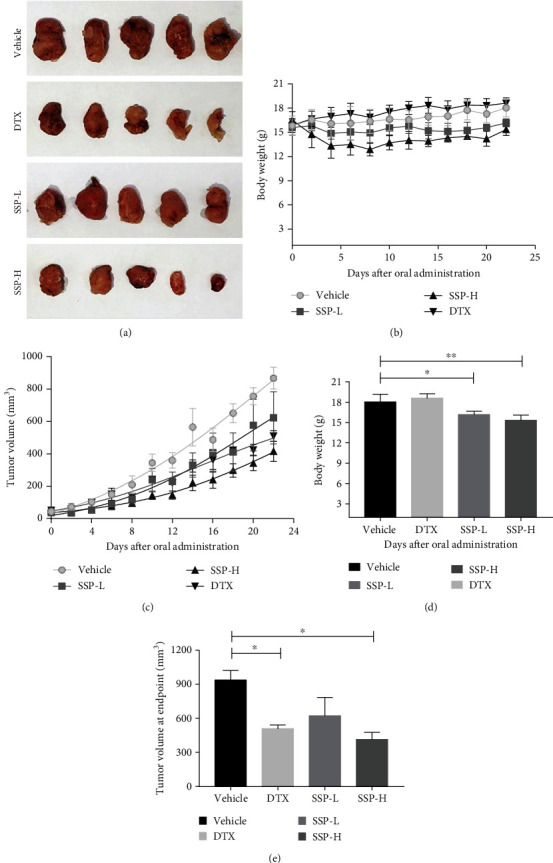
Effect of SSP on human TNBC cell line, MDA-MB-231, in the xenograft model. SSP played a positive role against MDA-MB-231. (a) Representative pictures of mice xenograft with different treatments for 22 days, (b) bodyweight curve, (c) tumor volume curve, and (d) analysis for the bodyweight of each group at an endpoint. Data were shown as mean ± SD (*n* = 5). (e) Analysis of tumor volume of each group at an endpoint. Data were shown as mean ± SE (*n* = 5). ^∗^*p* (vehicle vs.DTX) = 0.0435, ^∗^*p* (vehicle vs.SSP − H) = 0.0151.

Subcutaneous injections of MDA-MB-231 (2 × 10^6^ cells/0.1 ml) into the bilateral flank of each mouse were provided. Bodyweight, tumor growth, and tumor volume were monitored at 2 days intervals for 22 days. On day 22, the mice were sacrificed and measured their tumor volume at the endpoint (Figure 4(e)). There were significant body weight changes in the treated groups during the study period (Figure 4(b)). And treatment of SSP-L, SSP-H (0.4 and 0.8 g/kg/p.o, daily), and DTX (5 mg/kg/i.p. week) significantly inhibited the growth of tumors in the animals when compared to the vehicle group (Figures 4(c) and 4(e)). At the endpoint, tumor volume was significantly different (^∗^*p* = 0.0151) between the SSP-H-treated group and the vehicle group. In addition, DTX-treated animals were also significantly (^∗^*p* = 0.0435) reducing the growth of tumors (Figure 4(c)).

### 3.4. Evaluation of Acute Toxicity

The acute toxicity study was conducted to determine the harmful effects of SSP to the animals administered as a single or short-term exposure. This investigation assessed the changes in the behavior, sign, body weight, mortality, and other changes in the overall well-being of the animals. In the present study, the acute toxicity evaluation showed that the oral LD_50_ value of SSP was 10 g/kg b.w. (Table 1).

### 3.5. Generation of Intracellular ROS by Treatment with SSP in MDA-MB-231 Cell Lines

**Figure 5:**
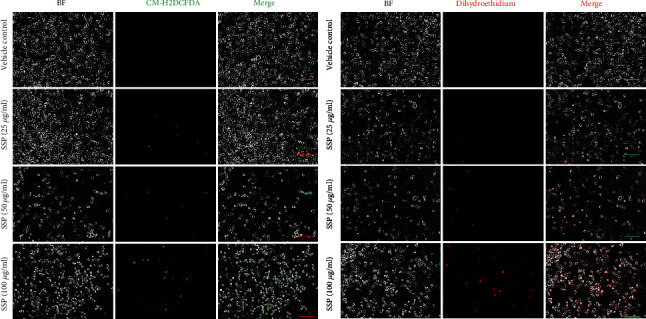
SSP upregulated ROS generation in MDA-MB-231 cells. The detection of intracellular ROS was based on CM-H2DCFDA and dihydroethidium staining of MDA-MB-231 cells after treatment with different doses of SSP (25, 50, and 100 *μ*g/ml) for 24 h. Scale bar, 100 *μ*m.

To determine the effect of SSP on ROS generation, ROS was detected using CM-H2DCFDA for general oxygen species and dihydroethidium staining, which facilitated to show the expression levels of superoxide and hydrogen peroxide. As shown in Figure 5, the Generation of ROS, specifically superoxide and hydrogen peroxide, were measured in a 24 h cultured plate containing MDA-MB-231 cells, which was shown in a dose-dependent manner of SSP (25, 50, and 100 *μ*g/ml). The outcomes showed that SSP treatment upregulated ROS generation in which the number of cells was stained using CM-H2DCFDA and dihydroethidium in MDA-MB-231 cells.

### 3.6. SSP Triggers Pyroptotic Cell Death in TNBC Cells

**Figure 6:**
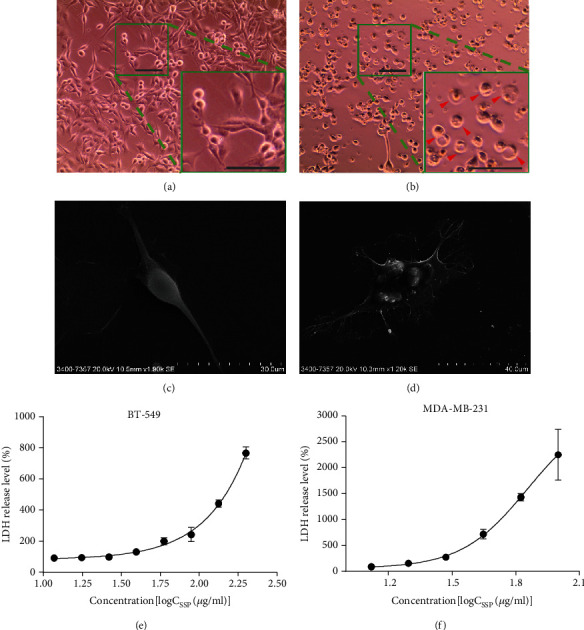
SSP promoted pyroptotic cell death in MDA-MB-231 cells. Bright field microscopic observation of (a, c) vehicle showed normal architecture of the cells. SEM observation (b, d) of SSP (100 *μ*g/ml) in MDA-MB-231 cells exhibited flattened cells with the cabbage or fried egg-like the cell nucleus located in the center. Arrowhead indicated the bubbling of pyroptotic cells. Scale bar, 100 *μ*m. (e, f) The release of LDH levels in BT-549 and MDA-MB-231 cell culture after 24 h treatment with SSP, respectively (100 *μ*g/ml).

**Figure 7:**
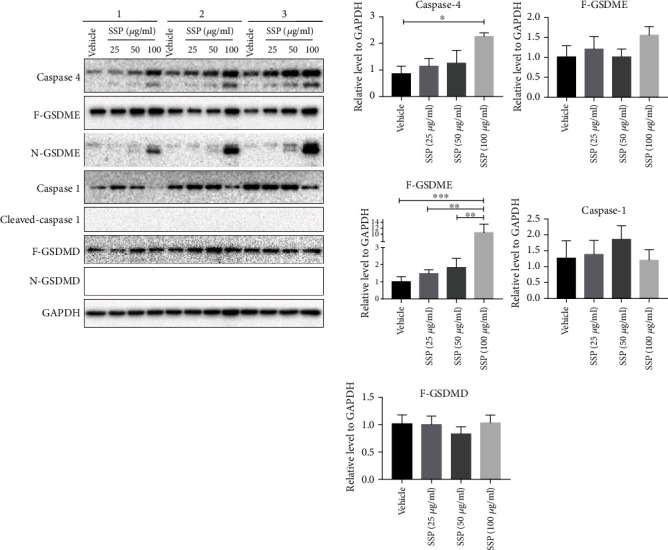
SSP triggered inflammatory cell death in MDA-MB-231 cells. Representative Western blot assay was involved in the pyroptotic signaling pathway in MDA-MB-231 cells that were connected with the treatment of SPP (25, 50, 100 *μ*g/ml). The expression of caspase-4 cleaved GSDME that triggers pyroptosis. The full-length GSDME (GSDME-F) degraded into N-terminal fragment of GSDME (N-GSDME) that directly transferred to the plasma membrane and lysed the cells (noncanonical pathway). However, caspase-1 did not involve in the cleaving of GSDMD-F (canonical pathway) in which there were no products of GSDMD-N, and hence this inflammasome mechanism was a ROS-dependent noncanonical pathway. Data were shown as mean ± SD (*n* = 3). ^∗∗^*p* = 0.0031, ^∗∗∗^*p* < 0.001.

The vehicle showed normal architecture of the MDA-MB-231 cells, which was observed under bright field by phase-contrast microscopy and SEM (Figures 6(a) and 6(c)). The treatment of SSP promoted pyroptotic cell death in 24 h cultures of MDA-MB-231 cells, which showed flattened cells with the cabbage or fried egg-like morphology in which the cell nuclei located in the center or above the main plane of the cell body (Figures 6(b) and 6(d)). The observations were well documented in the SSP-treated groups with noticeable pyroptotic features in the cell. During the pyroptotic mechanism, the activation of caspases causes the loss of membrane integrity and release of cytosolic LDH, resulting in inflammatory cell death. LDH with other cellular contents was discharged during pyroptotic blebs of cellular demise. The leakage of the LDH was determined in the culture medium using a commercial assay kit. Figures 6(e) and 6(f) exhibit the release levels of LDH in BT-549 and MDA-MB-231 cells after 24 h treatment with SSP (100 *μ*g/ml). The western blot analysis of inflammasome protein showed caspase-4 cleaved GSDME that permeabilized into the cell membrane and might trigger pyroptosis, a form of inflammatory programmed cell death (Figure 7). The full-length GSDME (F-GSDME) degraded into an N-terminal fragment of GSDME (N-GSDME) by caspase-4 that transported into the cell membrane and lysed the cells (Non-canonical pathway). Moreover, caspase-1 did not involve in the cleaving of GSDMD in which there were no products of GSDMD-N and therefore, this mechanism of the inflammasome was a ROS-dependent noncanonical pathway (Figure 7).

### 3.7. GSH Blocked SSP-Induced Pyroptosis in TNBC Cells

**Figure 8:**
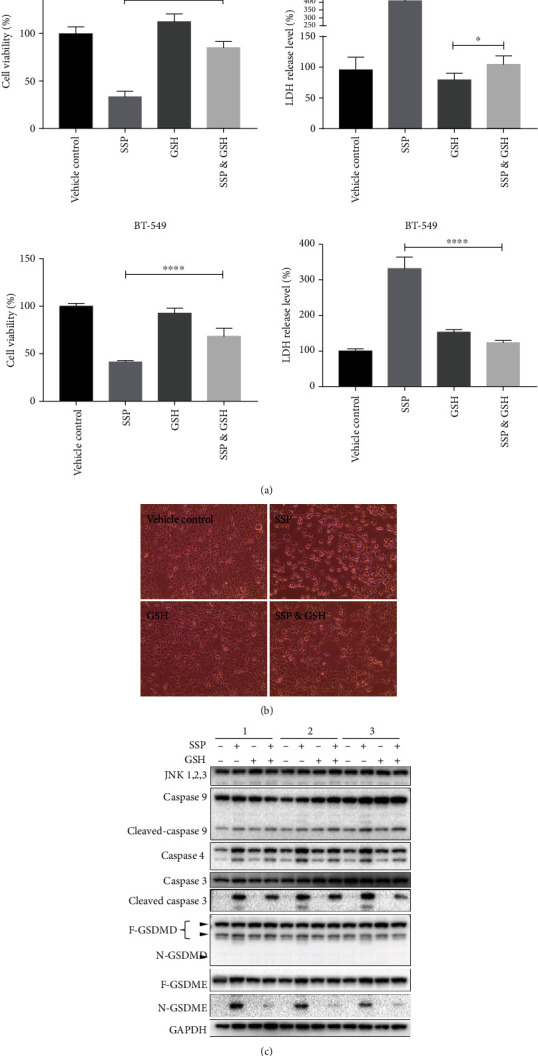
GSH blocked SSP-induced pyroptotic signaling pathways in TNBC cells. (a) Representative outcomes of the cell viability and LDH release assay in MDA-MB-231 and BT-549 cells upon the cotreatment of GSH and SSP. ^∗∗∗∗^*p* < 0.0001, ^∗^*p* = 0.0493. Data were shown as means ± SD (*n* = 6). (b) Representative phase-contrast microscopy of MDA-MB-231 cells upon the cotreatment of GSH and SSP. (c) Representative western blot assay for the detection of SSP induced pyroptotic inflammasome signaling pathways and GSH rescue experiment.

**Figure 9:**
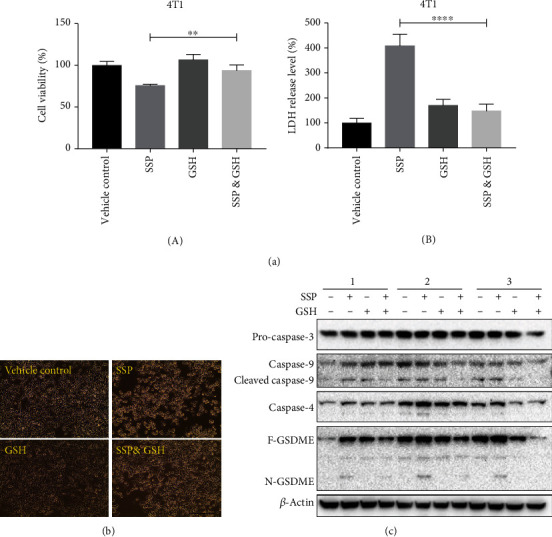
GSH blocked the efficacy of SSP and the pyroptotic signaling pathways in TNBC cells. (a) Representative result for cell viability detection of 4T1 cells after different treatments, ^∗∗^*p* < 0.01. Data are shown as means ± SD (*n* = 3). (b) Representative result of LDH release assay in 4T1 cells after different treatments, ^∗∗∗∗^*p* < 0.0001. Data are shown as means ± SD (*n* = 3). (b) Representative phase-contrast microscopy of 4T1 cells after different treatments. (c) Representative western blot assay for the detection of pyroptotic signaling pathways in GSH rescue experiment. BT-549 cells were pretreated with or without GSH (2 mM) for 2 h, followed by SSP (100 *μ*g/ml) or vehicle treatment for 24 h, respectively, as specifically indicated.

Glutathione (GSH) is an inhibitor of ROS, which markedly attenuates the SSP-induced ROS elevation in the cell and thus rescues pyroptotic cell death. BT-549 and MDA-MB-231 cells were pretreated with or without GSH (2 mmoles) for 2 h, followed by the treatment of SSP (100 *μ*g/ml) or vehicle (Milli-Q water) for 24 h. The pretreatment of GSH improved the cell viability and demolished the LDH release in SSP-treated MDA-MB-231 and 4 T1 cell lines due to its ROS scavenging potential (Figures 8(a) and 9(a)). Similarly, phase-contrast microscopic observation demonstrated that MDA-MB-231 and 4T1 cells treated with GSH followed by the administration of SSP showed less pyroptotic features, whereas SSP treatment showed flattened cells with fried egg-like morphology (Figures 8(b) and 9(b)). Western blot assay revealed GSH aborted pyroptotic signaling upon SSP treatment. Cleaved caspase-3, caspase-4, and GSDME were involved in the inflammasome signaling pathway (Figures 8(c) and 9(c)).

### 3.8. The Relationship between Caspase-4/9 and Overall Survival of the Patients

**Figure 10:**
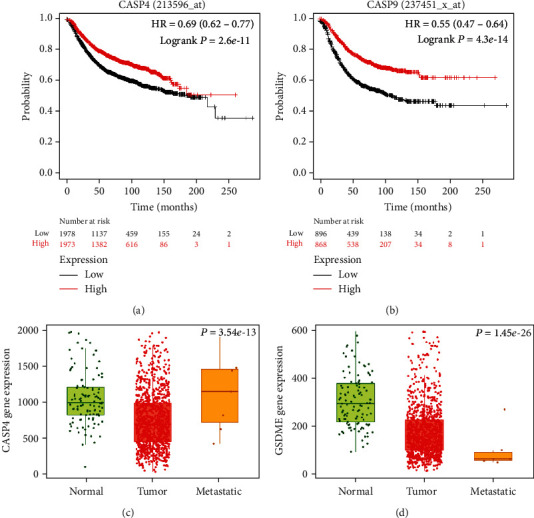
(a, b) The high expression of caspase-4/9 was connected with overall better survival of the BCa patients. (c, d) Analysis of GSDME and caspase-4 expression in normal, tumor, and metastatic states in BCa.

Confirming prognostic or projecting candidate genes in suitably powered BCa cohorts is of greatest interest nowadays. Based on the online Kaplan-Meier plotter tool, we drew survival plots, which were used to assess the relevant expression levels of caspase-4 and caspase-9 genes on the clinical outcome of BCs individuals. Using the selected parameters, the analysis was operated on caspase-4 (Affy ID: 233596, 3951 patients) and caspase-9 (Affy ID: 237451_x, 1751 patients). Based on the median of participants, the relevant expression levels were demonstrated at the lower or higher risks of 1978 and 1973, respectively. The hazardous ratio of caspase-4 was 0.69 (*p* value 1.5*e*-15) with the median months' survival of the respective low and high expression cohorts of 38 and 68.75. Similarly, caspase-9 was exhibited at lower and higher risks of 896 and 868, respectively. The hazardous ratio of caspase-9 was 0.55 (*p* value 4.3*e*-14) with the median months' survival of the respective low and high expression cohorts of 25.2 and 57. The high levels of the caspase-4 and caspase-9 expression in BCa patients were associated with better survival (Figures 10(a) and 10(b)).

### 3.9. Differential Gene Expression Analysis of GSDME and Caspase-4 in Tumor, Normal, and Metastatic Tissues

Genes generally show differential expression in either tumor or metastatic tissues, which can be beneficial to envisage tumor formation and to facilitate cancer management as a biomarker. Using the TNM plot tool, based on an integrated dataset that was documented in the RNA sequencing data of normal (*n* = 113), tumor (*n* = 1097), and metastatic (*n* = 07) tissues, we compared the differential expression levels of selected genes in normal, tumor, and metastatic tissues. The expression of caspase-4 and GSDME was significantly inhibited in the tumor tissues. The fold changes of caspase-4 from tumor to normal and from metastasis to the tumor were about 0.76 and 1.41, respectively (Figure 10(c)). The analysis of GSDME exhibited fold changes from tumor to normal (0.61) and from metastasis to tumor (1.14) (Figure 10(d)).

## 4. Discussion

*SSD* is a traditional medicinal plant normally used in China for its hematopoietic and antiviral properties [48, 49]. Mounting research has been conducted in vitro and in vivo of SSD showing as a promising traditional medicinal drug in the management of various cancers [15, 17, 20, 21]. Presently, physicians from TCM have utilized *SSD* as a potential therapy for BCa patients and accomplished greater positive outcomes [19]. Studies have further suggested that SSD directly suppresses various molecular signaling pathways, upregulates apoptotic signaling, inhibiting LDH and arresting the cell cycle, and is thereby proved as a potential anticancer compound [19, 21, 50]. In addition, SSD protects against various effects of oxidative stress, cerebral ischemia, radiation, and diabetic complications [51–53]. However, the anticancer efficacy of SSP and its protective mechanism against the most fetal and invasive subtype of BCa, TNBC have not been completely revealed.

Earlier, several studies were reported that SSD comprised of various bioactive compounds, viz., (-)-sativan, formononetin, isoliquiritigenin, genistein, naringenin, medicarpin, prestegane, naringenin, blumenol A, protocatechuic acid, liquiritigenin, 7,4′-dihydroxy-8-methoxy-isoflavone, protocatechuic acid, glycyroside, and dulcisflavan that possesses cytotoxicity, anticancer, and antimutagenic properties [7, 8, 13, 14, 19]. The present study is also exhibited five distinguished bioactive compounds, viz., catechin, procyanidin B2, epicatechin, genistein, and formononetin, and all of them have greater antitumor, antimutagenic, and potential genotoxic effects [31, 32, 54–59]. Some of these major compound(s) is/are responsible for the anti-TNBC efficacy of SSP, which is being studied in our laboratory. The outcome of acute toxicity indicated that the oral LD_50_ value of SSP was about 10 g/kg, and this extract is considered as low toxic to the animals. These findings offer preliminary data on the toxic profile of SSP. Hence, further studies (genotoxicity, subchronic toxicity, reproductive toxicity, etc.) are needed to validate the clinical studies of the plant.

In this study, SSP was investigated on three different TNBC cells: MDA-MB-231, 4T1, and BT 549 cell lines. SSP had significant cytotoxic and growth inhibitory effects on all cell lines in a dose-dependent manner. These effects can be mediated by the generation of ROS [60]. Previously, SSD treatment significantly increased cytotoxic effects through the generation of ROS in U266 and U937 cells [15]. ROS plays a critical role in multiple tumor chemotherapy and involves cytotoxicity, autophagy, and apoptosis [61]. There were significant differences in the SSP-treated groups (bodyweight-15.35 g and tumor volume-415.4 mm^3^) and vehicle group (bodyweight-18.04 g and tumor volume-937.4 mm^3^), which showed about 55.69% tumor growth inhibition at the endpoint. Thus, SSP inhibits the growth of MDA-MB-231 human TNBC in a xenograft-bearing mouse model. This study was consistent with the earlier study upon the treatment of resveratrol inhibited the gaining of body weight and tumor growth in the animal models [62, 63].

In our study, we detected ROS generation upon SSP treatment, which was consistent with earlier investigations [15, 17]. The detection of intracellular ROS was based on the presence of CM-H2DCFDA and dihydroethidium staining of MDA-MB-231 cells upon the treatment of SSP (25, 50, and 100 *μ*g/ml), and this ROS generation was significantly greater in MDA-MB-231 cells when treated with the higher concentrations of SSP (100 *μ*g/ml). For TNBC management, to date, there is no promising medication. Hence, there is urgent to find novel anti-TNBC strategies or molecular targets. Natural compounds like SSP, in this setting, have many advantages, especially in the clinical practices of TCM or in preclinical research. In our study, SSP could inhibit the growth of TNBC both in vitro and in vivo, and this mechanism has been elucidated through noncanonical pyroptotic pathways. The expression of caspase-4, cleaved caspase-9, GSDME, and the N-fragment of GSDME was upregulated upon SSP administration in TNBC cells.

Earlier, researchers believed only SSD treatment could induce apoptosis [15, 17]. However, in the present study, SSP promotes pyroptotic cell death in TNBC cells. Pyroptosis is a process of programmed cell death, mediated by the key factors, GSDMD or GSDME, which can be activated by caspase-4 and/or caspase-3 [64–66]. Several caspases can cleave GSDMD or GSDME into the N and C-terminal domain of GSDMD or GSDME, in which the N-terminal fragments have the ability of pore-forming activity in the plasma membrane [37, 67]. The significant difference between apoptosis and pyroptosis is the microscopy and cellular osmotic features. The morphological analysis of SSP-treated TNBC cells is of pyroptotic features. The cells are exhibited flattened cells with the “cabbage” or “fried egg”-like, and the cell nucleus located in the center. The activation of GSDME causes a loss of membrane integrity and release/discharge of cytosolic LDH, resulting in inflammatory cell death. LDH with other cellular contents is also discharged during the pyroptotic blebs of cellular demise [68]. Interestingly, SSP-treated TNBC cells have neither altered the expression of cleaved GSDMD nor cleaved caspase-1. This activation is performed through the activation of caspase-4 and caspase-3. The complex of N-GSDMEs inserts into the plasma membrane as pores resulting in cell lysis. In this process, canonical inflammasomes are not involved. Thus, the process was regarded as a noncanonical inflammasome pyroptotic signaling pathway.

The mechanisms underlying the events of the noncanonical inflammasome are still being described. Caspase-3, -8, -9, -7, -4, -5, and -11 trigger its activation, as they are recognized as molecular switches or effectors for pyroptotic cells [34, 65, 69–72]. These activated caspases then generate GSDME and biologically active executioners, impacting pyroptotic cell death [64]. GSH is an inhibitor of ROS that attenuates SSP-induced ROS generation in the cell and hence rescues pyroptotic cell death. SSP treatment alone could cause pyroptosis through the activation of caspase-4 and caspase-9. These findings indicated that SSP-induced pyroptotic death is ROS-dependent. The present investigation validated that SSP promotes ROS generation in TNBC cells which triggers noncanonical pyroptosis and involves a novel anti-TNBC-based intervention strategy for the treatment of BCa.

Noncanonical inflammasome-associated pyroptosis has been reported to play in both pro-and anti-tumor development. The tumor microenvironment is shaped by a chronic inflammation in which polarized macrophages and stromal components promote tumor development [73–75]. Thus, non-canonical inflammasome activation and regulation have been vital especially in cancer and other disease management. Non-canonical pro-pyroptotic agents like SSP induce an acute inflammatory immune response that warrants further investigation in a clinical setting. Furthermore, our bioinformatic analysis of caspase -4, -9, and GSDME are well-established as cancer markers that are also involving in non-canonical pyroptotic mechanisms.

Bioinformatic tools are extensively used to evaluate gene expression levels and to explore their possible implications in the development of various cancers [76–78]. In the present study, survival analysis using the KM Plotter revealed that the low transcription levels of caspase-4 and 9 are related to low relapse-free survival in BCa. This study was consistent with earlier investigations in which researchers concluded that the caspase family is operated as new prognostic indicators in various cancers, including breast [79], gastric [80], ovary [81], and renal [82]. TNM plot analysis showed that the expression of caspase-4 and GSDME was significantly inhibited in clinical tissues in normal (113), tumor (1097), and metastatic (07) states, which was consistent with the earlier investigation [47]. Based on the study, the ROS-induced pyroptotic pathway which is associated with caspase-4/9 and GSDME that are potential targets of precision therapy for patients with TNBC.

## 5. Conclusions

TNBC is one of the most problematic classes of BCa with easy recurrence and considerably assertive type. SSD has been used in the clinic of TCM as a potential therapeutic agent to heal BCa individuals, which accounts for relatively positive responses. However, the anti-TNBC potential of SSP and its evidence-based in vitro and preclinical studies is still deficient. Hence, the present study was evaluated the anti-TNBC potential of SSP through various in vitro and in vivo studies. SSP showed significant growth inhibitory efficacy in both TNBC cell lines and xenograft animal models. Western blot analysis was also encouraged that SSP elevated inflammasome proteins such as caspase-4 and 9, which cleaved GSDME triggering pyroptosis and permeabilizing the cell membrane. Furthermore, cotreatment of GSH and SSP markedly attenuates the SSP-induced ROS generation in the cell and validated the rescuing pyroptotic cell death. Survival analysis using the KM Plotter and TNM plot database exhibited the curved transcription levels of caspase-4 and 9 related to low relapse-free survival in patients with BCa. SSP is comprised of catechin, procyanidin B2, epicatechin, genistein, and formononetin that are recognized as anticancer agents. All findings strongly suggest that SSP possesses anti-TNBC efficacy and continues to be an inspiring and dynamic research niche in the upcoming days with evident antitumorigenesis effects and targets of eradicating BCa cells. However, well-controlled future clinical studies are quite required to advance an understanding of the pharmacological functions of SSP. Such information could be used to categorize effective preventive strategies targeting specific components of TNBC.

## Figures and Tables

**Table 1:** Mortality and clinical signs of acute toxicity of SSP.

Animals	Dose of SSP (g/kg b.w. p.o)	Mortality latency (h)	Toxic symptoms	LD_50_ (g/kg b.w. p.o.)
*BALB*/*c* mice	0	—	None	10
	2.0	—	None	
	4.0	—	None	
	8.0	—	None	
	10.0	<1 h	Anorexia, hypoactivity	

Sprague-Dawley rats	0	—	None	>10
	2.0	—	None	
	4.0	—	None	
	8.0	—	None	
	10.0	—	None	

Animals were divided into respective 5 groups and 6 animals (3 males and 3 females) each. Each group receiving a single oral dose of 2, 4, 8, and 10 g/kg body weight of SSP extract, while the control group was administrated with distilled water. The general behavior, signs of toxicity and/or death, and the latency of death were observed continuously for 1 h after the oral administration and then intermittently for 4 h and thereafter for 24 h. The LD_50_ value was determined according to the method described by Kharchoufa et al. [45].

## Data Availability

The data used to support the findings of this study are available from the corresponding author upon request.

## Abbreviations

AKT: Serine/threonine-specific protein kinase
ATCC: American Type Culture Collection
BCa: Breast cancer
BSA: Bovine serum albumin
CM-H2DCFDA: Chloromethyl derivative of 2′,7′-dichlorofluorescein
CULATR: Committee on the Use of Live Animals in Teaching and Research
DAD UV: Vis detector diode array detector ultraviolet-visible detector
DMEM: Dulbecco's Modified Eagle Medium
DTX: Docetaxel
ER+: Estrogen receptor
FBS: Fetal bovine serum
F-GSDME: Full-length GSDME
GAPDH: Glyceraldehyde 3-phosphate dehydrogenase
GSDMD: Gasdermin D
GSDME: Gasdermin E
GSH: Glutathione
H_2_O_2_: Hydrogen peroxide
IC_50_: Half maximal inhibitory concentration
LDH: Lactate dehydrogenase
MAPK: Mitogen-activated protein kinase
N-GSDME: N-Terminal fragment of GSDME
nmoles: Nanomoles
mmoles: Millimoles
PI3K: Phosphatidylinositol 3-kinase
RIPA: Radioimmunoprecipitation assay buffer
ROS: Reactive oxygen species
RPMI: Roswell Park Memorial Institute
SDS-PAGE: Sodium dodecyl sulphate–polyacrylamide gel electrophoresis
SEM: Scanning electron microscopy
SSD: *Spatholobus suberectus* Dunn
SSP: *Spatholobus suberectus* Dunn percolation extract
TNBC: Triple-negative breast cancer
UHPLC: Ultra high-pressure liquid chromatography.
